# A piezoelectric micro generator worked at low frequency and high acceleration based on PZT and phosphor bronze bonding

**DOI:** 10.1038/srep38798

**Published:** 2016-12-08

**Authors:** Gang Tang, Bin Yang, Cheng Hou, Guimiao Li, Jingquan Liu, Xiang Chen, Chunsheng Yang

**Affiliations:** 1 National Key Laboratory of Science and Technology on Micro/Nano Fabrication, Department of Micro/Nano Electronics, Shanghai Jiao Tong University, Shanghai, 200240, China; 2 Department of Mechanical and Engineering, Nanchang Institute of Technology, Nanchang, 330099, China

## Abstract

Recently, piezoelectric energy harvesters (PEHs) have been paid a lot of attention by many researchers to convert mechanical energy into electrical and low level vibration. Currently, most of PEHs worked under high frequency and low level vibration. In this paper, we propose a micro cantilever generator based on the bonding of bulk PZT wafer and phosphor bronze, which is fabricated by MEMS technology, such as mechanical chemical thinning and etching. The experimental results show that the open-circuit output voltage, output power and power density of this fabricated prototype are 35 V, 321 μW and 8664 μW cm^−3^ at the resonant frequency of 100.8 Hz, respectively, when it matches an optimal loading resistance of 140 kΩ under the excitation of 3.0 g acceleration. The fabricated micro generator can obtain the open-circuit stable output voltage of 61.2 V when the vibration acceleration arrives at 7.0 g. Meanwhile, when this device is pasted on the vibrating vacuum pump, the output voltage is about 11 V. It demonstrates that this novel proposed device can scavenge high vibration level energy at low frequency for powering the inertial sensors in internet of things application.

In recent years, energy harvesting has been investigated not only to response to the global energy shortage, but also to realize potential energy supply for micro-actuators, micro-sensors, especially the devices in civilian medical, and military fields. Wasted mechanical energy from ambient vibrations can be transformed into useful electrical power. Therefore, a variety of harvesters have been proposed to convert ambient energy into electrical energy under different mechanisms, including electromagnetic^1^,^2^,^3^,^4^,^5^, electrostatic^6^,^7^,^8^,^9^,^10^, triboelectric^11^,^12^,^13^,^14^,^15^,^16^,^17^ and piezoelectric^18^,^19^,^20^,^21^,^22^,^23^,^24^,^25^,^26^,^27^,^28^,^29^,^30^,^31^,^32^,^33^,^34^ mechanisms. However, the output power of these devices based on the first three mechanisms fabricated with complex fabrication processes cannot satisfy the power supply of electronic devices. A lot of piezoelectric energy harvesters (PEHs) specifically fabricated by MEMS technology^18^,^19^ have been explored recently due to its advantages of simple configuration and high output performance. Currently, most investigations for power generation of vibration-based PEHs focus on the configuration of cantilever unimorph beam with or without proof mass under *d*_31_ mode and *d*_33_ mode^20^. 5 μm PZT film for *d*_31_ and *d*_33_ modes MEMS generators was prepared by an aerosol deposition process^21^. In addition, a hydrothermal method^22^ and screen printing^23^ process have been developed for thick films over 5 μm in thickness. A sintering temperature larger than 550 °C can provide high piezoelectricity^24^, which limits the application of the following MEMS fabrication process. In order to improve the output performance of piezoelectric devices, bulk PZT bonding and thinning techniques have been developed^35^,^36^,^37^,^38^. In our previous work, the energy harvester of 14 μm PZT films was reported^39^, which was realized by the low temperature (175 °C) bonding and thinned-down techniques based on Si substrate. The mostly above reported piezoelectric energy harvesters based on silicon technology can be easily breaking down and only work at low vibration level from ambient sources due to the brittle properties of silicon. Currently, most of reported PEHs worked under high frequency. However, the frequencies of environmental vibration sources are relatively low (normally less than 200 Hz)^40^. For example, a small microwave oven works at the resonant frequency of 121 Hz, a washing machine at 109 Hz, a kitchen blender casing at 121 Hz and a CD on notebook computer at 75 Hz^40^. Liu *et al*. demonstrated a low-frequency silicon piezoelectric energy harvester, which generated the output power from 19.4 nW to 51.3 nW within the operation frequency bandwidth ranging from 30 Hz to 47 Hz at 1.0 g^41^. Then a S-shaped MEMS micro generator with 2.5 μm PZT film deposited by sol-gel process was reported, which generated the power of 42 mV at low frequency (<30 Hz) at 0.06 g^42^. Although fewer papers have reported low-frequency piezoelectric energy harvesters, they worked below 2.0 g. Moreover, their output power is very low. However, in the fields of aerospace and military, the working acceleration of the ballistic missile and rocket during launching can reach from 4 g to15 g. Meanwhile, in our environment, the acceleration of automobile and hand tool also can arrive at 10.0 g^43^,^44^,^45^,^46^,^47^,^48^. Phosphor bronze as an alloy of copper has good electrical conductivity and resistance to fatigue, so the phosphor bronze can replace silicon as the substrate of the energy harvester worked at the low frequency and high level vibration.

Here in this paper, we have developed a micro piezoelectric generator based on bulk PZT bonding on the phosphor bronze composite structure to convert vibration energy into electricity, which can realize a low resonant frequency and scavenge the vibration energy at the high acceleration of 7.0 g. The key techniques for fabricating micro generators are described, including the bulk PZT-Cu-Si bonding, bulk PZT thinning and patterning. In order to reduce the resonant frequency of this device, apart from the integration of silicon proof mass, the mass of tungsten is assembled on the end of top surface from the composite cantilever. This proposed energy harvester has lightened nine LED bulbs in series. Moreover, it can effectively scavenge vibration energy from the vacuum pump.

## Results

### Structure and Fabrication

**Figure 1:**
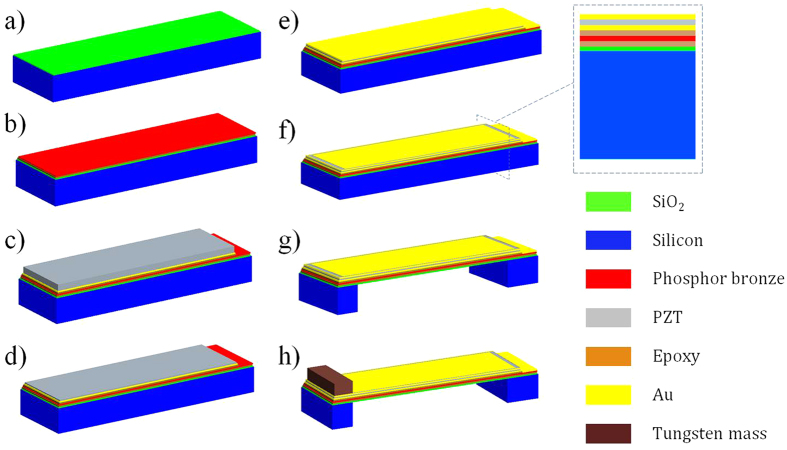
Fabrication process of micro generator. (**a**) Silicon oxide deposited on silicon wafer. (**b**) Bonding phosphor bronze and silicon wafer and thinning down the phosphor bronze by the mechanical lapping. (**c**) Bulk PZT with bottom electrode bonding with phosphor bronze. (**d**) Thinning down PZT by using the mechanical lapping and wet-etching combined method and polishing. (**e**) Top electrode deposition. (**f**) Top electrode patterning. (**g**) The backside etching by DRIE. (**h**) Tungsten mass assembling at the end of the cantilever.

**Figure 2:**
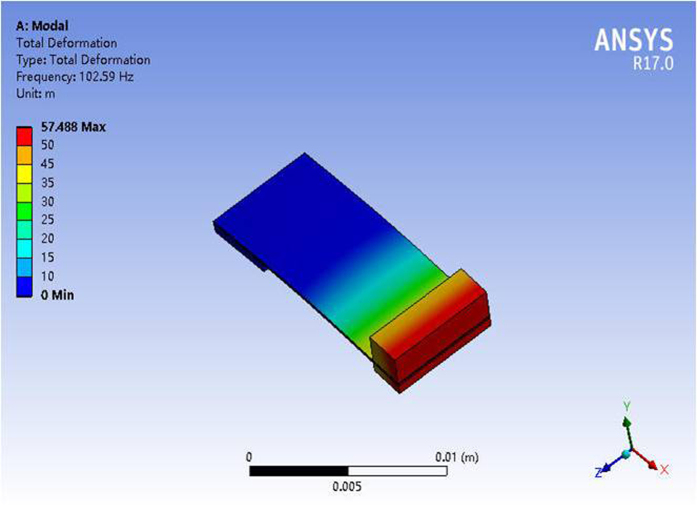
Finite element simulation of the designed device.

Figure 1 illustrates the fabricating process of micro designed generator. The whole structure includes phosphor bronze as supporting layer, bulk PZT thin film as functional piezoelectric layer, conductive epoxy as low temperature bonding layer and proof mass. The started wafer of 400 μm silicon with 2 μm silicon oxide layer is used to bond with 100 μm thick phosphor bronze, as shown in Fig. 1(a). The phosphor bronze should be thinned to the appropriate thickness for reducing the resonant frequency and the top surface should be polished to improve the bonding force with bulk PZT, as shown in Fig. 1(b). Then, one side of 400 μm thick PZT is polished and deposited with the 20 nm/180 nm Cr/Au bottom electrode layer. This side of bulk PZT was bonded together with phosphor bronze by epoxy resin, as shown in Fig. 1(c). PZT layer was thinned down to the required thickness by means of the mechanical lapping and wet-etching combined method, as shown in Fig. 1(d). Figure 1(e) and (f) illustrate the Cr/Au layer as the top electrode was sputtered and patterned by dry etching to form the top and bottom electrode, respectively. Figure 1(g) shows that the handle Si layer was etched from the backside to release the cantilever with silicon proof mass structure by deep reactive ion etching (DRIE) process. Finally, Fig. 1(h) shows that the rectangle tungsten proof mass cut by dicing method is assembled on the top of the cantilever. The designed dimension of the piezoelectric micro generator is listed in Table 1. The resonant frequency of this device is simulated by finite element software of ANSYS. The frequency of the first resonant mode is calculated as about 102.59 Hz, as shown in Fig. 2.

**Figure 3:**
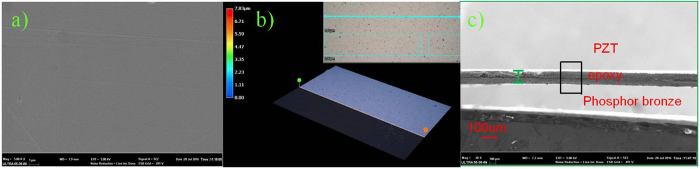
Micro fabricated piezoelectric images of microscope. (**a**) SEM image of the PZT surface after thinning and polishing. (**b**) 3-D digital microscope of the PZT surface after thinning and polishing. (**c**) Cross-sectional view of the composite cantilever.

**Figure 4:**
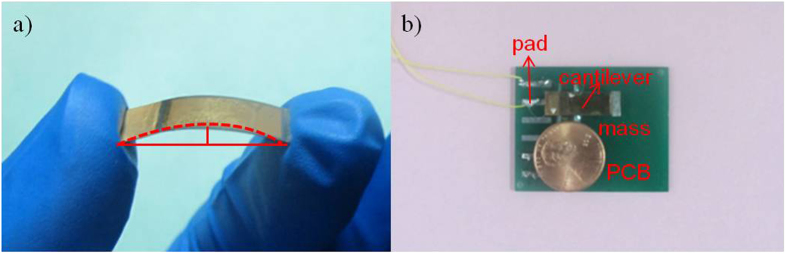
Micro fabricated piezoelectric cantilever and assembled with PCB. (**a**) The bending status of the fabricated piezoelectric cantilever. (**b**) Micro fabricated piezoelectric device assembled with PCB.

**Figure 5:**
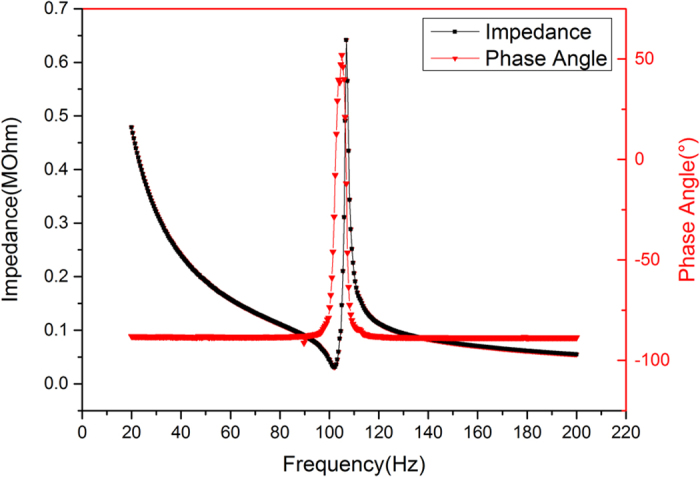
Measured resonant frequency of a typical device using the impedance analyzer.

Generally, it is difficult to thin down the ceramic plate to less than 50 μm due to its brittleness. Therefore, the hybrid method of mechanical lapping and wet-etching processes is deployed to thin down 400 μm thickness bulk PZT to about 57 μm by mechanical lapping. An additional mechanical polishing process on PZT film was applied to improve the surface roughness of PZT for subsequent fabrication processes. Moreover, good surface roughness contributes to improve the adhesive force of following deposited metal layer. SEM image of the surface the PZT after grinding and polishing technique is shown in Fig. 3(a). It is seen that there are no any big holes in Fig. 3(b). The roughness of about 0.82 μm is measured by digital microscope. Figure 3(c) shows the cross-sectional view with the high-quality bonding interface after thinning down PZT. The conductive epoxy resin between PZT and phosphor bronze can be seen clearly. The thickness of the PZT layer after thinning is uniform throughout the diaphragm. Figure 4(a) shows the bending status of the micro fabricated cantilever under the small compression force applied by fingers. It demonstrates that the composite cantilever based on the bonding of PZT and phosphor bronze wafer has a good flexibility. Then, the fabricated device is fixed on PCB with an acrylic spacer of 2000 μm thickness to make this device freely move even at high acceleration level. The top and bottom electrodes on PZT functional layer are connected with two bonding pads in PCB, respectively, as shown in Fig. 4(b). Figure 5 shows the measured phase angle of the fabricated device versus the exciting frequencies using an impedance analyser (KEYSIGHT E4990A). The peak of the phase-angle curve is at about 105 Hz. There is a good agreement between theoretical and experimental results.

### Output Performance

**Figure 6:**
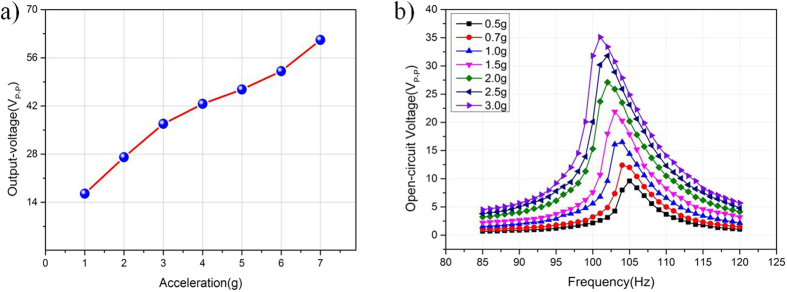
Open-circuit output voltage. (**a**) Open-circuit output voltage from 1 g to 7.0 g at resonant frequency. (**b**) The output open-circuit voltages of the harvester when it is excited under different accelerations from 0.5 g to 3.0 g.

In order to investigate the output performance of the fabricated generator, the corresponding output average power was calculated by the following equation:


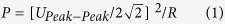


where *U*_*peak*−*Peak*_ is the loading AC peak-peak voltage of close circuit, and *R* is the loading resistance. Figure 6(a) shows the output maximum open-circuit voltages versus resonant vibration frequency at different vibration levels. It is observed that the output voltages of the micro fabricated generator at 1.0 g, 2.0 g, 3.0 g, 4.0 g, 5.0 g, 6.0 g, and 7.0 g acceleration are 16.5 V, 27.1 V, 36.8 V, 42.6 V, 46.8 V, 52.1 V, and 61.2 V, respectively. The maximum output voltage increases with increasing vibration acceleration value. It is found that at high input acceleration of 7.0 g, the output voltage of is still stable. In order to obtain its output performance of the generator in detail, the output voltages at the range of 85 Hz to 120 Hz are conducted at the accelerations from 0.5 g to 3.0 g. As seen from Fig. 6(b), for input accelerations of 0.5 g, 0.7 g, 1.0 g, 1.5 g, 2.0 g, 2.5 g, 3.0 g, the open circuit output voltages can achieve 10 V, 12.4 V, 16.5 V, 21.7 V, 26.3 V, 30.4 V, 35 V at the frequencies of 104.9 Hz, 104.4 Hz, 104.0 Hz, 103.2 Hz, 102.3 Hz, 101.9 Hz, 100.8 Hz, respectively, which gradually decrease with the increase of the acceleration amplitude. The difference is mainly because of the nonlinear change in Young’s modulus of PZT^39^.

**Figure 7:**
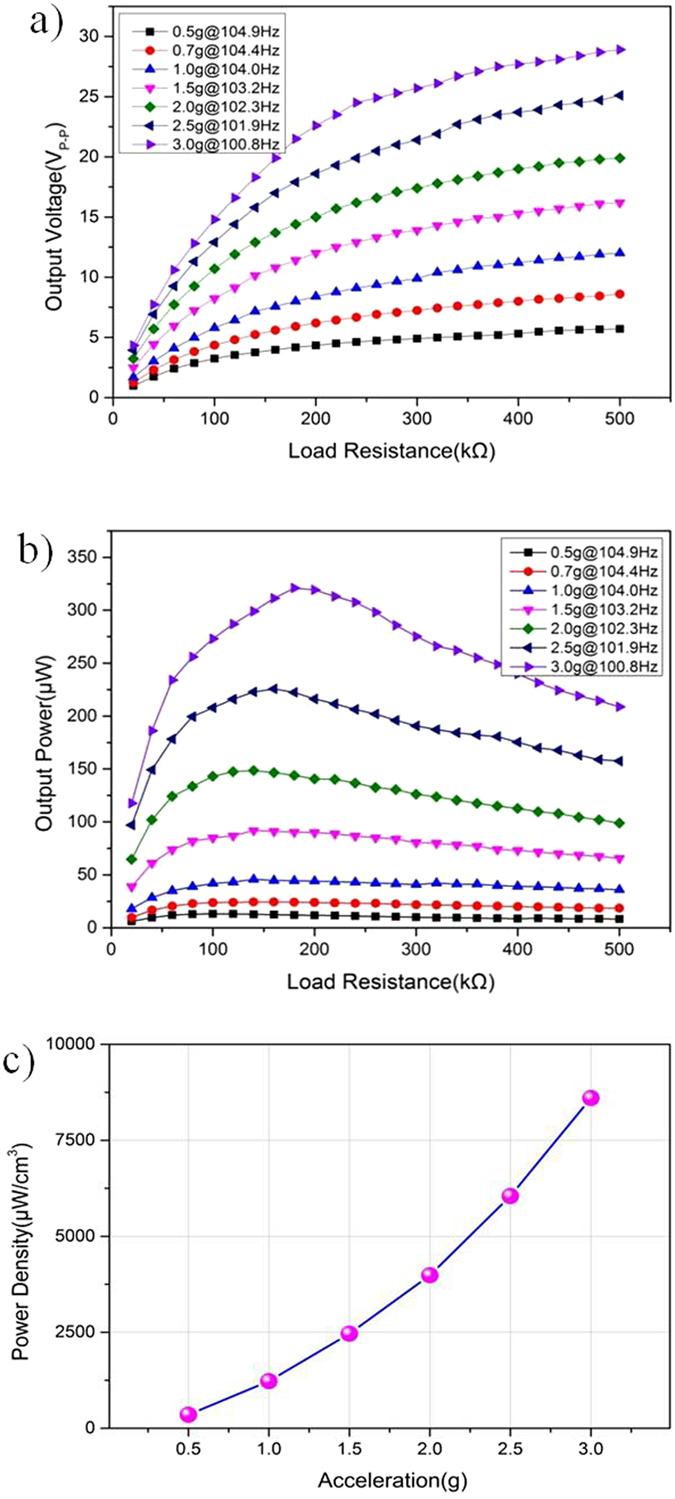
Loading output voltage, power and power density versus the loading resistance at different accelerations. (**a**) The output voltage under different loading resistances. (**b**) The output power under different loading resistances. (**c**) The power density at resonant frequency from 0.5 g to 3.0 g.

When the resonant vibration was achieved, the output power of the micro generator was highly dependent on the loading resistance and the maximum output power can be obtained under certain loading resistance. Figure 7(a) shows the output voltage at different loading resistance under different excited accelerations from 0.5 g to 3.0 g. Under a certain acceleration exciting condition, the output voltage increased with increasing loading resistance. The increasing trend at the range of lower matching resistance is larger than that under the condition of higher resistance. Under each excitation conditions, the voltage output curve trends to level and eventually starts close to the open circuit voltage output value. Based on equation (1), the corresponding output power is shown in Fig. 7(b). With the increase of loading resistance, the output power firstly increased and then decreased at certain acceleration. There is a peak value of output power under each excited acceleration condition at a certain load resistance, which is named the optimal-matched resistance. When the loading resistance is matched with the internal resistance of the micro generator, the maximum output powers are 13.1 μW, 24.4 μW, 45.8 μW, 91.9 μW, 148.6 μW, 225.8 μW and 321 μW at 0.5 g, 0.7 g, 1.0 g, 1.5 g, 2.0 g, 2.5 g and 3.0 g, respectively.

In order to compare the output performance of the fabricated micro generator with different micromachining processes of bonding PZT with phosphor bronze wafer, the output power density is deployed to be evaluated. The effective volume of the fabricated micro generator is calculated as 0.03732 cm^3^, along with the acceleration from 0.5 g to 3.0 g, 24.7 times the power density increased, from 351.02 μW cm^−3^ to 8664 μW cm^−3^, as shown in Fig. 7(c). Compared to the power density reported by^39^, this device has good output performance working under high acceleration. Some reported devices operated in the frequency range of 10 Hz to 300 Hz are listed in Table 2 for comparison. This indicates that the power density of the piezoelectric device reported in this paper demonstrates comparable performance at low frequency range. However, the thickness of phosphor bronze and bulk PZT can be reduced further to shrink the whole dimension for improving the output power density.

## Discussion

**Figure 8:**
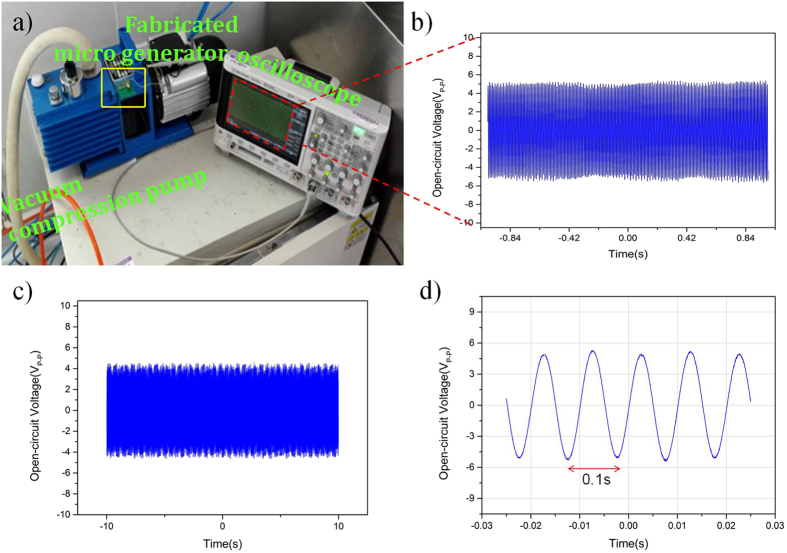
The output voltage of the fabricated micro generator tested on the vacuum compression pump. (**a**) The photograph of the fabricated micro generator prototype tested on the vacuum compression pump. (**b**) The output voltage in 2 s. (**c**) The output voltage in 20 s. (**d**) The output voltage in 0.05 s.

To validate the capacity of the fabricated micro generator in practical application, the micro generator was tested on vacuum pump with the rotation speed of 1400 r min^−1^ (type 2XZ-2), which was used in vacuum drying oven. Figure 8(a) shows the photograph of the fabricated micro generator prototype fixed on the pump and an oscilloscope (Agilent DSO-X) was used to record the output voltage. When the vacuum pump works, the vibration of the pump drives the cantilever of the device to oscillate. Subsequently, the micro generator will generate electrical voltage due to the oscillation of the cantilever. Figure 8(b) shows the output voltage of the micro generator fixed on the pump. The open-circuit output voltage arrives at 11 V_p-p_ in 2 s. As shown in Fig. 8(c), the output electrical signal of this generator is stable and sustainable in 20 s, which can be used to power a lot of micro electrical equipments or charge the battery. Figure 8(d) shows the output voltage is stable with wave period of 0.01 s. Therefore, the working frequency of this device is calculated as approximately 100 Hz, which is close to the resonant frequency of this generator.

**Figure 9:**
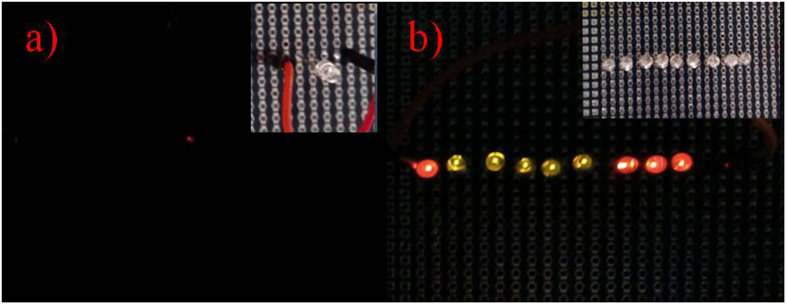
Application of the fabricated micro generator. (**a**) The fabricated micro generator tested on the vacuum compression pump is lighting 1 LED bulb. (**b**) The fabricated micro generator is lighting 9 LED bulbs at the acceleration of 3.0 g.

**Figure 10:**
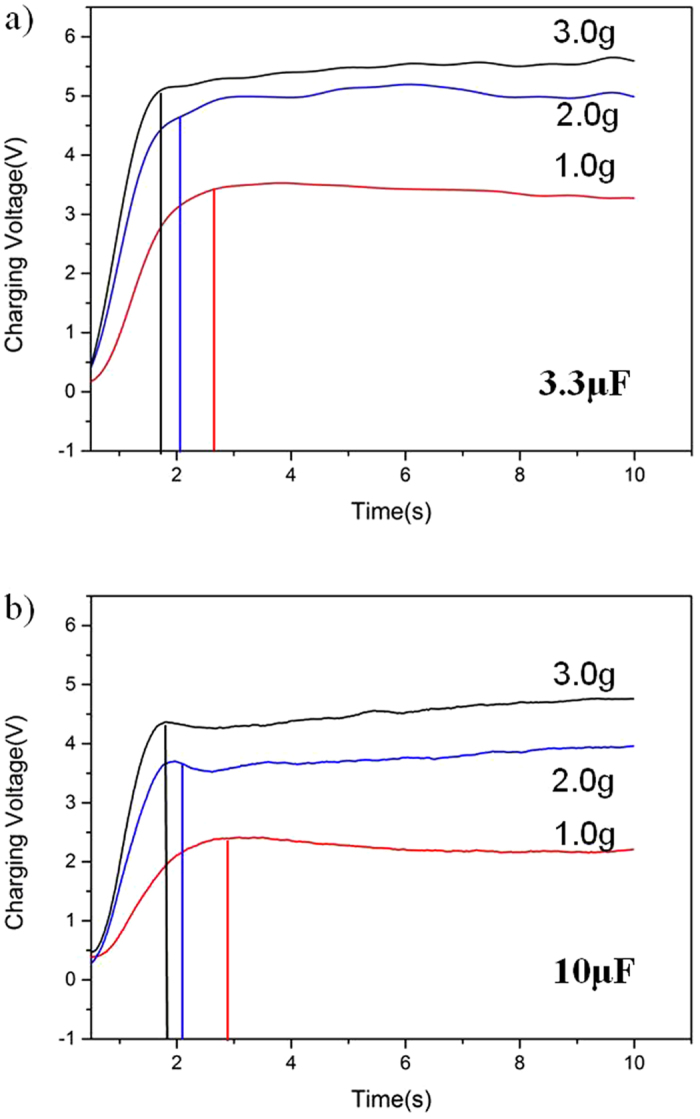
Charging tests of 3.3 μF and 10 μF from 1.0 g to 3.0 g. (**a**) Charging voltage and time of 3.3 μF with different vibration accelerations. (**b**) Charging voltage and time of 10 μF with different vibration acceleration.

Meanwhile, the micro generator can light LED bulbs under working on the vacuum pump and the vibration acceleration of 3.0 g. Figure 9(a) shows that one light emitting diode (LED) bulb is lighten up by the micro generator worked on the vacuum pump due to its vibration level. While we put this device on the shaker with the acceleration 3.0 g, as shown in Fig. 9(b), this generator lighten nine LED bulbs configured in series. Figure 10 shows the diagram of the charging voltages and times of two capacitors with 3.3 μF and 10 μF at 1.0 g, 2.0 g, and 3.0 g, respectively. By using a full-wave rectifier made by four diodes, at the acceleration of 1.0 g, the micro generator can charge 3.3 μF and 10 μF capacitors to 3.6 V and 2.4 V after 2.3 s and 2.4 s, respectively. With the increasing of vibration acceleration, the chargeable voltage becomes higher and the time needs less. When the acceleration increases to 3.0 g, 3.3 μF and 10 μF capacitors arrive at 5.2 V and 4.5 V after 1.8 s and 1.9 s, respectively. It is concluded that the charging time of the capacitor with 3.3 μF capacitor is much faster than that of 10 μF. With the advanced development of micro-nano fabrication technologies and microelectronics circuits, some devices with low-power consumption are developed to work for a long time. Low power consumption of sensors has quickly developed, such as the wireless body temperature sensor system controlled by a microcontroller unit with a power consumption of 27 μW in active mode^49^. The working voltage of some monitoring sensors such as the digital tri-axial vibration sensor is 3.0 V. This confirmed that this developed micro generator can provide power autonomy for some low consumption electronic devices.

In summary, this paper has demonstrated the design, fabrication, and characterization of a novel MEMS-based piezoelectric energy harvester device based on PZT and phosphor bronze bonding technique, which operates at a low frequency and high acceleration vibration. In this design, a tungsten proof mass is assembled with the cantilever beam of phosphor bronze and piezoelectric wafer to realize a resonant frequency as low as 105 Hz. Due to the elasticity and fatigue resistance effect of the phosphor bronze, the device can withstand vibration acceleration of 7.0 g and generate the output voltage of 61.2 V. As a result, for input accelerations from 0.5 g to 3.0 g, the output voltages are from 10 V to 35 V. In the case of 3.0 g acceleration, the output power of the micro generator could reach 321 μW. Due to the high output performance, the micro generator could lighten one LED tested on the vacuum compression pump and nine LEDs at the acceleration of 3.0 g. This device can be applied in the environments of low-frequency and high-level vibrations, such as aerospace and military fields.

## Methods

### Bonding and thinning process of composite structure

Phosphor bronze wafer (thickness of 100 μm) was bonded with silicon wafer first, then thinned to the required thickness and polished to add adhesive force with the followed PZT wafer. Finally, the polished bulk PZT (thickness of 400 μm) was bonded with the prepared wafer with conductive epoxy by facing each other. The bonded wafer was put in a vacuum environment, and with 0.1 MPa pressure and the curing temperature constant at 175 °C for 3 hours.

### Method of output performance testing

The output performance of the micro generator was evaluated by a vibration testing system. It consists of an electromagnetic shaker (SINOCERA JZK-5), a power amplifier (SINOCERA YE2706A), a function waveform generator (Agilent 33220A), and an accelerometer (SINOCERA YE5932A). The fabricated micro generator and the accelerometer were assembled onto the shaker during experiments. The vibration frequency and amplitude of the shaker were controlled by the function generator and the power amplifier, and the vibration conditions were monitored by the accelerometer. The output signal of the device was connected with different loading resistances to optimize the output power at different vibration conditions. The output voltages from the testing sample were recorded by the oscilloscope (Agilent DSO-X).

## Additional Information

**How to cite this article**: Tang, G. *et al*. A piezoelectric micro generator worked at low frequency and high acceleration based on PZT and phosphor bronze bonding. *Sci. Rep.* **6**, 38798; doi: 10.1038/srep38798 (2016).

**Publisher's note:** Springer Nature remains neutral with regard to jurisdictional claims in published maps and institutional affiliations.

## Supplementary Material

Supplementary Information

Supplementary Video

## Figures and Tables

**Table 1:** Dimension of the designed micro generator.

Structural layers	Geometrical parameters (μm)	Value
Phosphor bronze	Length	12000
	Width	6000
	Thickness	57
PZT	Length	11000
	Width	5000
	Thickness	57
Tungsten mass	Length	5500
	Width	1920
	Thickness	2400
Silicon mass	Length	6000
	Width	1920
	Thickness	4400
epoxy	Thickness	4

**Table 2:** Performance comparison among published piezoelectric harvesters operated at low frequency range.

Author	Input vibration (g)	Frequency (Hz)	Power output (μW)	Power density (μWcm^−3^)
Lee *et al*.^21^	1.0	255.9	1.125	2647
Aktakka *et al*.^36^	1.5	154	205	11417
Liu H *et al*.^32^	0.8	25	0.01	159.4
Shen *et al*.^50^	0.75	183.8	0.32	416
Lei *et al*.^51^	1.0	235	14.0	1086
Tang *et al*. (this work)	3.0	100.8	321	8664

## References

[b1] Beeby S. P. . A micro electromagnetic generator for vibration energy harvesting. J. Micromech. Microeng. 17, 1257–1265 (2007).

[b2] Chen Y., Pollock T. E. & Salehian A. Analysis of compliance effects on power generation of a nonlinear electromagnetic energy harvesting unit: theory and experiment. Smart Mater. Struct. 22, 094027 (2013).

[b3] Sari I., Balkan T. & Kulah H. An electromagnetic micro power generator for wideband environmental vibrations. Sens. Actuators A. 145, 405–413 (2008).

[b4] Bai X. L. . A magnetoelectric energy harvester with the magnetic coupling to enhance the output performance. J. Appl. Phys. 111, 07A938 (2012).

[b5] Glynne-Jones P., Tudor M. J., Beeby S. P. & White N. M. An electromagnetic vibration-powered generator for intelligent sensor systems. Sens. Actuators A 110, 344–349 (2004).

[b6] Yang B., Lee C., Kotlanka R. K., Xie J. & Lim S. P. A MEMS rotary comb mechanism for harvesting the kinetic energy of planar vibrations. J. Micromech. Microeng. 20, 065017 (2010).

[b7] Zhu G., Wang A. C., Liu Y., Zhou Y. & Wang Z. L. Functional electrical stimulation by nanogenerator with 58 V output voltage. Nano Lett. 12, 3086–3092 (2012). 22594588 10.1021/nl300972f

[b8] Pelrine R. . Dielectric elastomers: generator mode fundamentals and applications. Proc. SPIE 4329, 148–156 (2001).

[b9] Miao P. . MEMS inertial power generators for biomedical applications. Microsyst. Technol. 12, 1079–1083 (2006).

[b10] Wang S., Xie Y., Niu S., Lin L. & Wang Z. L. Freestanding triboelectric-layer-based nanogenerators for harvesting energy from a moving object or human motion in contact and non-contact modes. Adv. Mater. 26, 2818–2824 (2014). 24449058 10.1002/adma.201305303

[b11] Lin L. . Segmentally structured disk triboelectric nanogenerator for harvesting rotational mechanical energy. Nano Lett. 13, 2916–2923 (2013). 23656350 10.1021/nl4013002

[b12] Zhu G., Peng B., Chen J., Jing Q. & Wang Z. Triboelectric nanogenerators as a new energy technology: From fundamentals, devices, to addplications. Nano Energy 14, 126–138 (2015).

[b13] Bai P. . Cylindrical rotating triboelectric nanogenerator. ACS Nano 7, 6361–6366 (2013). 23799926 10.1021/nn402491y

[b14] Zheng Q. . *In Vivo* Self-Powered Wireless Cardiac Monitoring Via Implantable Triboelectric Nanogenerator. ACS Nano. 10, 6510–6518 (2016). 27253430 10.1021/acsnano.6b02693

[b15] Cheng . Wearable electrode-free triboelectric generator for harvesting biomechanical energy. Nano Energy. 12, 19–25 (2015).

[b16] Pi Z., Zhang J., Wen C., Zhang Z. & Wu D. Flexible piezoelectric nanogenerator made of poly (vinylidenefluoride-co-trifluoroethylene) (PVDF-TrFE) thin film. Nano Energy. 7, 33–41 (2014).

[b17] Zhu Y. B. . A flexible and biocompatible triboelectric nanogenerator with tunable internal resistance for powering wearable devices. Sci. Rep. 6, 22233 (2016). 26916819 10.1038/srep22233 PMC4768091

[b18] Kamal T. M. . Modeling and characterization of MEMS-based piezoelectric harvesting devices. J. Micromech. Microeng. 20, 105023 (2010).

[b19] Park J. C., Park J. Y. & Lee Y. P. Modeling and characterization of piezoelectric d33-mode MEMS energy harvester. J. Microelectromech. 19, 1215–1222 (2010).

[b20] Qin Y., Wang X. & Wang Z. L. Microfibre-nanowire hybrid structure for energy scavenging. Nature 451, 809–813 (2008). 18273015 10.1038/nature06601

[b21] Lee B. S. . Piezoelectric MEMS generators fabricated with an aerosol deposition PZT thin film. J. Micromech. Microeng. 19, 065014 (2009).

[b22] Kanda T., Kurosawab M. K., Yasui s. H. & Higuchi T. Performance of hydrothermal PZT film on high intensity operation. Sens. Actuators A 89, 16–21 (2001).

[b23] Beeby S. B., Blcakburn A. & White N. M. Processing of PZT piezoelectric thick films on silicon for micro electromechanical systems. J. Micromech. Microeng. 9, 218–229 (1999).

[b24] Wang X. Y., Lee C. Y., Peng C. J., Chen P. Y. & Chang P. Z. A micrometer scale and low temperature PZT thick film MEMS process utilizing an aerosol deposition method. Sens. Actuators A 143, 469–474 (2008).

[b25] Li Z., Zhu G., Yang R., Wang A. C. & Wang Z. L. Muscle-driven *in vivo* nanogenerator. Adv. Mater. 22, 2534–2537 (2010). 20446305 10.1002/adma.200904355

[b26] Shi B. . A Packaged Self-Powered System with Universal Connectors Based on Hybridized Nanogenerators. Adv. Mater. 28, 846–852 (2016). 26634808 10.1002/adma.201503356

[b27] Shi Q., Wang T. & Lee C. MEMS Based Broadband Piezoelectric Ultrasonic Energy Harvester (PUEH) for Enabling Self-Powered Implantable Biomedical Devices. Sci. Rep. 6, 24946 (2016). 27112530 10.1038/srep24946 PMC4844957

[b28] Shi Q., Wang T., Kobayashi T. & Lee C. Investigation of geometric design in piezoelectric microelectromechanical systems diaphragms for ultrasonic energy harvesting. Appl. Phys. Lett. 108, 193902 (2016).

[b29] Liu H., Zhang S., Kobayashi T., Chen T. & Lee C. Flow sensing and energy harvesting characteristics of a wind-driven piezoelectric Pb (Zr0.52, Ti0.48) O3 microcantilever. Micro & Nano Lett. 9, 286–289 (2014).

[b30] Liu H., Zhang S., Kathiresan R., Kobayashi T. & Lee C. Development of piezoelectric micro cantilever flow sensor with wind-driven energy harvesting capability. Appl. Phys. Lett. 100, 223905 (2012).

[b31] Dhakar L., Liu H., Tay F. E. H. & Lee C. A new energy harvester design for high power output at low frequencies. Sens. Actuators A 199, 344–352 (2013).

[b32] Liu H., Lee C., Kobayashi T., Tay C. J. & Quan C. Piezoelectric MEMS-based wideband energy harvesting systems using a frequency-up-conversion cantilever stopper. Sens. Actuators A 186, 242–248 (2012).

[b33] Liu H., Lee C., Kobayashi T., Tay C. J. & Quan C. Investigation of a MEMS piezoelectric energy harvester system with a frequency-widened-bandwidth mechanism introduced by mechanical stoppers. Smart Mater. Struct. 21, 035005 (2012).

[b34] Liu H., Tay C. J., Quan C., Kobayashi T. & Lee C. A scrape-through piezoelectric MEMS energy harvester with frequency broadband and up-conversion behaviors. Microsyst. Technol. 17, 1747–1754 (2011).

[b35] Tanaka K., Konishi T., Ide M. & Sugiyama S. Wafer bonding of lead zircon tetitanate to Si using an intermediate gold layer for micro device application. J. Micromech. Microeng. 16, 815 (2006).

[b36] Aktakka E. E., Peterson R. L. & Najafi K. Thinned-PZT on SOI process and design optimization for piezoelectric inertial energy harvesting. Transducer’s 11, 1649–1652 (2011).

[b37] Xu X. H. & Chu J. R. Preparation of a high-quality PZT thick film with performance comparable to those of bulk materials for applications in MEMS. J. Micromech. Microeng. 18, 065001 (2008).

[b38] Xu X. H., Li B. Q., Feng Y. & Chu J. R. Design, fabrication and characterization of a bulk-PZT-actuated MEMS deformable mirror. J. Micromech. Microeng. 17, 2439 (2007).

[b39] Tang G. . Fabrication and analysis of high-performance piezoelectric MEMS generators. J. Micromech. Microeng. 22, 065017 (2012).

[b40] Roundy S., Wright P. K. & Rabaey J. A study of low level vibrations as a power source for wireless sensor nodes. J. Comput. Commun. 26, 1131–1144 (2003).

[b41] Liu H., Tay C. J., Quan C., Kobayashi T. & Lee C. Piezoelectric MEMS energy harvester for low-frequency vibrations with wideband operation range and steadily increased output power. J. Micromech. Microeng. 20, 1131–1142 (2011).

[b42] Liu H., Lee C., Kobayashi T., Tay C. J. & Quan C. A new S-shaped MEMS PZT cantilever for energy harvesting from low frequency vibrations below 30 Hz. Microsyst. Technol. 18, 497–506 (2012).

[b43] Roundy S. On the effectiveness of vibration-based energy harvesting. J. Intel Mat. Syst. Str. 16, 809–823 (2005).

[b44] Roundy S. & Wright P. K. A piezoelectric vibration based generator for wireless electronics. Smart Mater. Struct. 13, 1131–1142 (2004).

[b45] Bottner H. . New thermoelectric components using microsystem technologies. J. Microelectro. Mech. S. 13, 414–420 (2004).

[b46] Ikeda K., Ishizuka H., Sawada A. & Urushiyama K. Vibration acceleration magnitudes of hand-held tools and work pieces. Industrial Health 36, 197–208 (1998). 9583318 10.2486/indhealth.36.197

[b47] Frechin M. M., Arino S. B. & Fontaine J. Actiseat: Active vehicle seat for acceleration compensation. P. I. Mech. Eng. D-J. 218, 925–933 (2004).

[b48] Fang H. B. . Fabrication and performance of MEMS-based piezoelectric power generator for vibration energy harvesting. J. Microelectron. 37, 1280–1284 (2006).

[b49] Zhong J. . Fiber-based generator for earable electronics and mobile medication. ACS Nano. 8, 6273–6280 (2014). 24766072 10.1021/nn501732z

[b50] Shen D. . Micromachined PZT cantilever based on SOI structure for low frequency vibration energy harvesting. Sens. Actuators A 154, 103–108 (2009).

[b51] Lei A. . MEMS-based thick film PZT vibrational energy harvester. In Micro Electro Mechanical Systems (MEMS), *2011 IEEE 24th International Conference*. 125–128 (2011).

