# Supplementary material for: A piezoelectric micro generator worked at low frequency and high acceleration based on PZT and phosphor bronze bonding
Source: Sci Rep. 2016 Dec 8;6:38798. doi: 10.1038/srep38798 (PMC5144009; doi:10.1038/srep38798)
Supplement: Supplementary Information [file srep38798-s1.pdf]

A piezoelectric micro generator worked at low frequency and high acceleration based on PZT and phosphor bronze bonding

Gang Tang[1, 2], Bin Yang[1,*], Cheng Hou[2], Guimiao Li[1], Jingquan Liu[1], Xiang Chen[1] and Chunsheng Yang[1]

[1] *National Key Laboratory of Science and Technology on Micro/Nano Fabrication, Department of Micro/Nano Electronics, Shanghai Jiao Tong University, Shanghai, 200240, China.*
[2] *Department of Mechanical and Engineering, Nanchang Institute of Technology, Nanchang, 330099, China*

Corresponding Author: Bin Yang

E-mail: *binyang@sjtu.edu.cn*

Video captions

Video 1 9 LED bulbs are lighting

The fabricated micro generator is lighting 9 LED bulbs at the acceleration of 3.0 g.
